# PD-L1 expression on immune cells, but not on tumor cells, is a favorable prognostic factor for head and neck cancer patients

**DOI:** 10.1038/srep36956

**Published:** 2016-11-14

**Authors:** Hye Ryun Kim, Sang-Jun Ha, Min Hee Hong, Su Jin Heo, Yoon Woo Koh, Eun Chang Choi, Eun Kyung Kim, Kyoung Ho Pyo, Inkyung Jung, Daekwan Seo, Jaewoo Choi, Byoung Chul Cho, Sun Och Yoon

**Affiliations:** 1 Yonsei Cancer Center, Division of Medical Oncology, Yonsei University College of Medicine, Seoul, Korea; 2 Department of Biochemistry, College of Life Science & Biotechnology, Yonsei University, Seoul, Korea; 3 Department of Otorhinolaryngology, Yonsei University College of Medicine, Seoul, South Korea; 4 Department of Pathology, Yonsei University College of Medicine, Seoul, Korea; 5 JE-UK Institute for Cancer Research, JEUK Co., Ltd., Gumi-City, Kyungbuk, Korea; 6 Department of Biostatistics and Medical Informatics, Yonsei University College of Medicine, Seoul, Korea; 7 Severance Biomedical Science Institute, Yonsei University of College of Medicine, Seoul, Korea

## Abstract

To investigate the expression of programmed death-ligand 1 (PD-L1) and immune checkpoints and their prognostic value for resected head and neck squamous cell cancer (HNSCC). PD-L1 expression on tumor cells (TC) and tumor-infiltrating immune cells (IC), abundance of tumor-infiltrating lymphocytes (TILs), and expression of the immune checkpoints were investigated in 402 HNSCC patients. PD-L1 expression on TC and IC was categorized into four groups according to the percentage of PD-L1-positive cells. PD-L1 positivity was defined as ≥5% of cells based on immunohistochemistry. High PD-L1 expression on IC, but not TC, was an independent favorable prognostic factor for RFS and OS adjusted for age, gender, smoking, stage, and HPV. High frequencies of CD3^+^ or CD8^+^ TILs, Foxp3^+^ T_reg_s, and PD-1^+^ TILs were strongly associated with favorable prognosis. PD-L1 was exclusively expressed on either TC or IC. Transcriptome analysis demonstrated that IC3 expressed higher levels of the effector T cell markers than TC3, suggesting that PD-L1 expression is regulated via an adaptive IFNγ-mediated mechanism. High PD-L1 expression on IC, but not TC, and high abundance of PD-1^+^ T cells and Foxp3^+^ T_reg_s are favorable prognostic factors for resected HNSCC. This study highlights the importance of comprehensive assessment of both TC and IC.

The prognosis of recurrent or metastatic head and neck squamous cell cancer (R/M HNSCC) patients is dismal, as their median overall survival (OS) is approximately six to nine months^1^. Programmed cell death protein-1 (PD-1) is an immune inhibitory receptor that interacts with two ligands, programmed death ligand 1 (PD-L1) and programmed death ligand 2 (PD-L2)^1^,^2^,^3^. PD-L1 is widely expressed on antigen-presenting cells and other immune cells (IC)^1^,^2^,^4^ and is upregulated on tumor cells (TC) from a broad range of cancer types, including HNSCC. The PD-1/PD-L1 interaction is a major immune checkpoint that has been implicated in the adaptive immune resistance of HNSCC^1^,^2^,^3^. Recently, there has been a breakthrough in cancer immunotherapy against various cancer types by employing immune checkpoint blockade, particularly using antibodies directed against PD-1/PD-L1 pathway members^3^,^5^. Anti-PD-1 or anti-PD-L1 blockade is expected to hold promise as a treatment of patients with HNSCC based on the preliminary results of clinical trials using pembrolizumab or durvalumab (MEDI4736), which demonstrated that PD-L1 expression might be a predictive biomarker for the efficacy of anti-PD-1/PD-L1 blockade^2^,^6^,^7^. Pembrolizumab demonstrated an overall response rate (ORR) of 20%, and subgroup analysis showed a higher response rate among those positive for PD-L1 (45.5%) than among those negative for PD-L1 (11.4%)^7^. Similarly, durvalumab displayed an ORR of 14% in R/M HNSCC patients, with a response rate of 50% among those whose tumors displayed PD-L1 positivity but only 6% among those whose tumors displayed PD-L1 negativity^6^. Currently, various phase III clinical trials testing pembrolizumab (NCT02252042 and NCT02358031), nivolumab (NCT02105636), and the combination of durvalumab and tremelimumab (NCT02369874) in R/M HNSCC patients are underway.

The immune system plays a key role in the development, establishment, and progression of HNSCC, and various mechanisms of immune evasion by the tumor have been found in this form of cancer^8^. The presence of CD8^+^ tumor-infiltrating lymphocytes (TILs) has been known to predict the response of solid tumors to anti-PD1 therapy^9^,^10^. During immune evasion by HNSCC, various immune checkpoints may be exploited by the immune system in the tumor microenvironment to induce the exhaustion of effector T cells. Several receptors, including cytotoxic T lymphocyte associated protein-4 (CTLA-4), lymphocyte-activation gene-3 (LAG-3), T cell immunoglobulin mucin protein-3 (TIM-3), and PD-1, have been identified on exhausted and dysfunctional lymphocytes and are upregulated in cell lines of multiple tumor types, including HNSCC^8^,^11^. CTLA-4, a member of the B7 receptor family, is expressed by CD4^+^ T cells, CD8^+^ T cells, and regulatory T cells (T_reg_s) and competes with CD28 to bind to the stimulatory ligands CD80 and CD86^8^,^12^. LAG-3 is another receptor that has been shown to enhance T_reg_ function^8^,^13^. The role of TIM-3 as a marker or mediator of immunosuppression is still under investigation, but studies have correlated TIM-3 expression levels with poor clinical outcomes^8^,^14^. Identification of the expression profile and prognostic impact of these molecules has facilitated further establishment of immunotherapies^8^. At present, the prognostic value of PD-L1 is still controversial, and the distribution of PD-L1 on TC or IC has not been comprehensively analyzed in HNSCC. PD-L1 expression has been correlated with poor clinical outcome in several cancer types, although PD-L1 has been associated with improved survival and increased TIL frequency in some reports^15^,^16^,^17^,^18^,^19^,^20^,^21^,^22^. Therefore, determining the frequency and prognostic impact of these molecules in HNSCC patients can aid in the development of drugs for this cancer type.

Here, we analyzed the distribution of CD3^+^/CD8^+^ T cells and T_reg_s and the expression of immune checkpoints, such as PD-1, ICOS, and LAG-3, on IC. Moreover, we comprehensively quantified PD-L1 expression on TC and IC and examined whether the PD-L1 status, the presence of infiltrating T cells, and the expression of immune checkpoints was associated with the clinical and pathological features or survival outcomes of HNSCC patients. Ultimately, we aimed to determine the prognostic value of PD-L1 and various immune checkpoints in HNSCC patients to identify their potential therapeutic relevance.

## Patients and Methods

### Patients

This study was conducted on a cohort of HNSCC patients who underwent surgical resection at Severance Hospital in Seoul, Korea, between 2005 and 2012. The patient selection criteria included (1) surgically resected HNSCC with a curative aim, (2) availability of tumor tissue and clinical data on smoking status and survival outcomes, (3) no preoperative treatment, and (4) no distant metastasis. We excluded 121 patients whose samples had undergone decalcification. Ultimately, the tumor samples of 402 patients were available for examination of immune markers. Tumor location and size, histologic grade, metastasis to regional lymph nodes, lymphovascular invasion, and perineural invasion were evaluated. Tumors were classified according to the Seventh American Joint Committee on Cancer (AJCC) TNM cancer classification system and the World Health Organization classification system. Two experienced pathologists (S.O.Y. and E.K.K.), who were blinded to the clinical data for the patients, confirmed the diagnosis of HNSCC based on hematoxylin and eosin (H&E) staining. A predesigned data collection format was used to review the patients’ medical records for evaluation of clinicopathological characteristics and survival outcomes. This study was approved by the Institutional Review Board of Severance Hospital and all experiments were performed in accordance with relevant guidelines and regulations. We obtained informed consent from all subjects.

### Tissue microarray preparation, immunohistochemistry (IHC), and analysis

Sections of formalin-fixed paraffin-embedded tissues were prepared and stained with H&E. Under a microscope, representative tumor areas were confirmed and selected to generate tissue microarrays (TMAs). Two or three different representative tumor areas per sample were selected, and tissue cores (3 mm in diameter) were collected from the individual FFPE blocks and arranged in recipient paraffin blocks (TMA blocks) using a trephine. All TMA blocks were confirmed to contain suitable tumor lesions representing more than 50% of the core area based on H&E staining.

IHC was performed on 4-μm TMA tissue sections using a Ventana Bench Mark XT Autostainer (Ventana Medical Systems, Tucson, AZ, USA). Primary antibodies against the following antigens were used: PD-L1 (dilution 1:100; clone SP142; Ventana), PD-1 (dilution 1:100; clone NAT105; Cell Marque, Rocklin, CA, USA), CD3 (dilution 1:200; LabVision, Fremont, CA, USA), CD8 (RTU; clone C8/144B; Dako, Glostrup, Denmark), Foxp3 (dilution 1:100; Abcam, Cambridge, UK), ICOS/CD278 (clone SP98; dilution 1:50; Thermo Scientific, Rockford, IL, USA), LAG-3 (clone EPR4392(2); dilution 1:100; Abcam), and p16 (RTU; Ventana).

### Immunohistochemical analysis

PD-L1 expression on TC and IC was analyzed separately by applying a modification of a previously used approach^2^. IC were defined as T cells and macrophages/dendritic cells that infiltrated into tumor cell nests (intraepithelial immune cells)^2^. PD-L1 expression by TC and IC was categorized into four groups according to the percentage of PD-L1-positive cells: TC0 or IC0, 0%; TC1 or IC1, >0% but <5%; TC2 or IC2, ≥5% but <50%; and TC3 or IC3, ≥50%. PD-L1 positivity was defined as PD-L1 expression on ≥5% of all TC or IC based on immunohistochemistry. The densities of TILs were semi-quantitatively scored by measuring CD3^+^ and CD8^+^ T cells based on previously used methods^23^,^24^,^25^. The abundance of TILs infiltrating tumor cell nests was evaluated by examining five representative high-power fields under 400x magnifications. Preserved intact lymphocytes expressing CD3 or CD8 were counted manually, and the cell counts were averaged. The frequencies of PD-1-, Foxp3-, ICOS-, and LAG-3-positive TILs were measured using the same method as mentioned above. PD-1 positivity was defined as PD-1 expression on ≥5% of all TILs. For CD3^+^, CD8^+^, ICOS^+^ and LAG-3^+^ T cells and Foxp3^+^ T_reg_s, if the cell density was above the median value, the sample was defined as high density for that cell type. For p16 IHC, conventionally accepted criteria were used, and samples were determined to be positive for p16 when strong and diffuse nuclear and cytoplasmic staining was observed in more than 70% of all HNSCC tumor cells. All other staining patterns were scored as negative^26^,^27^.

### Transcriptome analysis

A total 10 ng of RNA was reverse-transcribed using the Ion AmpliSeq Transcriptome Human Gene Expression Kit according to the manufacturer’s protocol (Life Technologies, Carlsbad, CA, USA). The Ion Proton reads were analyzed using the AmpliSeqRNA analysis plugin, v4.2.1, in Torrent Suite Software (Life Technologies, Carlsbad, CA, USA). This program counts the number of sequences obtained for all cDNA amplicons. The resulting counts represent the gene expression levels for over 20,800 different genes present in the AmpliSeq Human Gene Expression panel^28^.

### Statistical analysis

The correlations between immune markers and patient characteristics were analyzed using the chi-square test with χ^2^ correction or Fisher’s exact test for categorical variables. Survival variables were estimated using the Kaplan–Meier method and compared via the log-rank test for categorical variables or a Cox regression model and the associated Wald chi-square statistic for quantitative variables. Overall survival (OS) was defined as the time from the initial diagnosis until death or the most recent follow-up. Relapse-free survival (RFS) was measured from the time of surgery to initial tumor relapse (local or distant recurrence) or death from any cause. Patients with no signs of relapse were censored at the time of the most recent follow up or death. The median follow-up duration for the overall population was 46.3 months.

## Results

### Baseline clinicopathological data

The cohort of 402 primary HNSCC tumor samples was evaluated for PD-L1 expression on TC and IC as well as for PD-1, LAG-3, and ICOS expression on IC. The baseline clinicopathological characteristics of the patients are presented in Table 1. The median age was 58 years (range 22–88 years), and the majority of the patients were males (75.1%) and either current or ex-smokers (61.1%). The predominant primary tumor sites were the oral cavity (204/402, 50.7%) and the oropharynx (122/402, 30.3%). The percentages of patients at each pathological stage were 28.9% at stage I, 10.7% at stage II, 18.4% at stage III, and 42.0% at stage IVA/B. Over the median follow-up duration of 46.3 months, 81 (20.1%) patients had relapsed, and 74 (18.4%) patients had died. The 5-year RFS and OS rates were 71.9% and 79.6%, respectively. As expected, survival outcomes were well differentiated according to the disease stage (Supplementary Fig. 1). We analyzed whether TIL abundance or PD-L1 expression correlated with the clinicopathological features. No significant correlation of TIL frequency with T stage, N stage or smoking history was observed. The expression of PD-L1 on TC or IC was not significantly correlated with any clinicopathological or basic characteristics.

### High TIL abundance is a favorable prognostic factor for HNSCC patient survival

**Figure 1:**
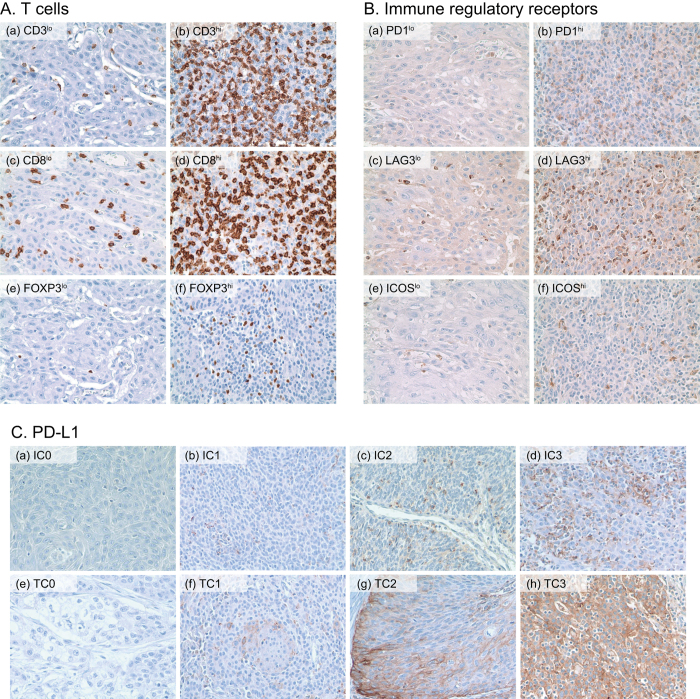
(**A**) A representative sample showing CD3^+^, CD8^+^, and regulatory T cells: (a) low infiltration of CD3^+^ T cells (b) high infiltration of CD3^+^ T cells (c) low infiltration of CD8^+^ T cells (d) high infiltration of CD8^+^ T cells (e) low infiltration of Foxp3^+^ T_reg_s (f) high infiltration of Foxp3^+^ T_reg_s. (**B**) A representative sample showing the expression of immune regulatory receptors on immune cells: (a) low expression of PD-1 (b) high expression of PD-1 (c) low expression of LAG-3 (d) high expression of LAG-3 (e) low expression of ICOS (f) high expression of ICOS. (**C**) PD-L1 expression was semi-quantitatively graded in tumor-infiltrating immune cells (IC) as IC0, 0% (a); IC1, >0% but <5% (b); IC2, ≥5% but <50% (c); and IC3 ≥50% (d). In tumor cells (TC), PD-L1 expression was graded as TC0, 0% (e); TC1, >0% but <5% (f); TC2, ≥5% but <50% (g); and TC3, ≥50% (h).

**Figure 2:**
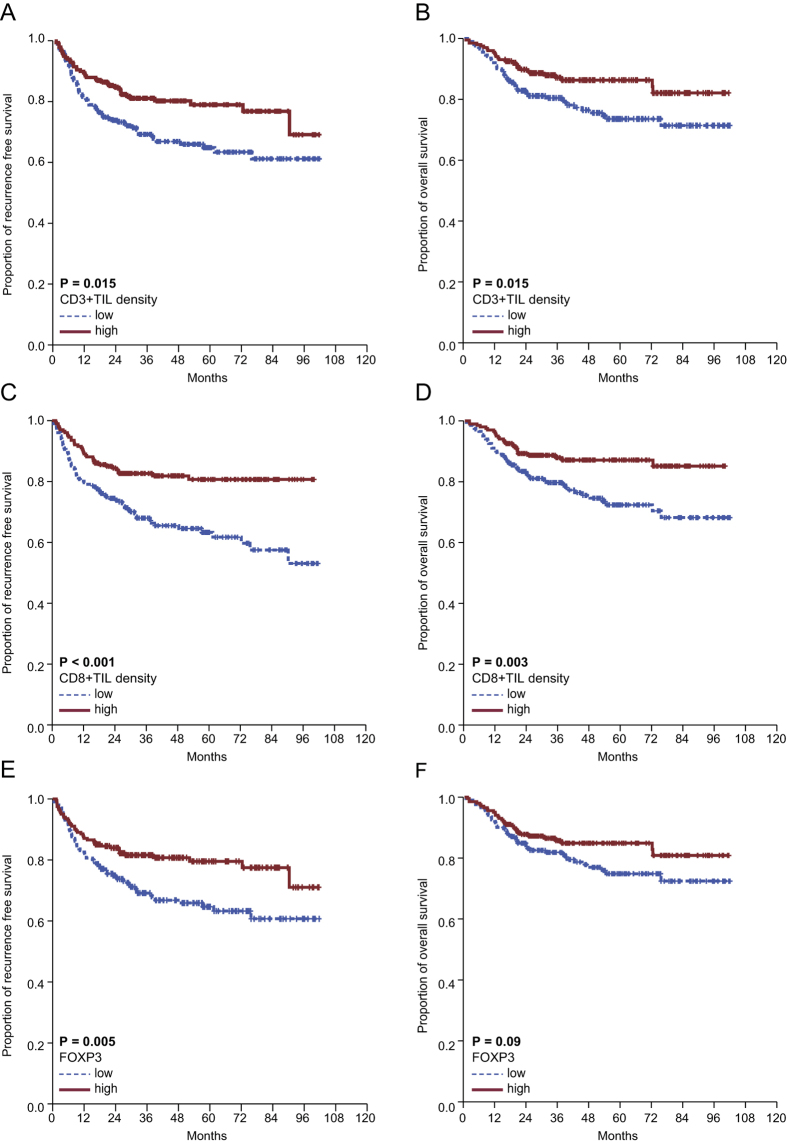
The 402 included patients were assigned to one of two groups based on specific subsets of T lymphocytes. (**A**) The 5-year RFS rate was 65.0% for low CD3^+^ vs. 79.1% for high CD3^+^; *P* = 0.005. (**B**) The 5-year OS rate was 73.5% for low CD3^+^ vs. 86.3% for high CD3^+^; *P* = 0.01. (**C**) The 5-year RFS rate was 63.3% for low CD8^+^ vs. 80.7% for high CD8^+^; *P* < 0.0001. (**D**) The 5-year OS rate was 72.4% for low CD8^+^ vs. 87.1% for high CD8^+^; *P* = 0.003. (**E**) The 5-year RFS rate was 64.7% for low Foxp3 T_reg_ vs. 79.5% for high Foxp3 T_reg_; *P* = 0.05. (**F**) The 5-year OS rate was 74.8% for low Foxp3 T_reg_ vs. 84.9% for high Foxp3 T_reg_; *P* = 0.09.

All patients were classified into two groups: a low or high abundance of TILs, according to the median abundances of CD3^+^ TILs (36/HPF) and CD8^+^ TILs (24/HPF) (Fig. 1A(a–d)). The specific frequencies of CD3^+^, CD8^+^, and Foxp3^+^ TILs are presented in Supplementary Fig. 2. Patients with a high frequency of CD3^+^ TILs demonstrated significantly higher 5-year rates of RFS (79.1% vs. 65.0%) and OS (73.5% vs. 86.3%) than those with a low frequency of CD3^+^ TILs. Univariate analysis revealed a low risk of disease relapse (hazard ratio [HR]: 0.57, 95% CI: 0.38–0.84, *P* = 0.005) and death (HR: 0.56, 95% CI: 0.35–0.90, *P* = 0.01) among patients with a high frequency of CD3^+^ TILs (Fig. 2A,B). Patients with high CD8^+^ TIL counts also demonstrated significantly higher 5-year rates of both RFS (80.7% vs. 63.3%) and OS (87.1% vs. 72.4%) than those with low CD8^+^ TIL counts (Fig. 2C,D). Multivariate analysis showed that patients with a high abundance of CD3^+^ TILs had significantly lower risks of recurrence and death than those with a low abundance of CD3^+^ TILs after adjusting for sex, smoking, age, pathologic stage, and HPV infection (RFS: adjusted HR [AHR] 0.57, 95% CI 0.39–0.8, *P* = 0.005; OS: AHR 0.56, 95% CI 0.35–0.90, *P* = 0.017) (Supplementary Table 1). A high abundance of CD8^+^ TILs also correlated with a significantly lower risk of recurrence and death than a low abundance of CD8^+^ TILs after adjusting for sex, smoking, age, pathologic stage, and HPV infection (RFS: AHR 0.46, 95% CI 0.31–0.70, *P* < 0.001; OS: AHR 0.48, 95% CI 0.30–0.78, *P* = 0.003) (Supplementary Table 1).

Patients were stratified into two groups according to the median abundance of Foxp3^+^ T cells (7/HPF), referred to as the low and high TIL cohorts (Fig. 1A(e,f)). A high abundance of Foxp3^+^ T_reg_s was strongly associated with a superior survival outcome relative to a low abundance of Foxp3^+^ T_reg_s. Kaplan-Meier curves highlighted the poorer survival outcomes of patients with a low Foxp3^+^ T_reg_ count than of those with a high Foxp3^+^ T_reg_ count (5-year RFS rate: 79.5% vs. 64.7%, *P* = 0.05; 5-year OS rate: 84.9% vs. 74.8%, *P* = 0.09) (Fig. 2E,F).

### PD-1, LAG-3, and ICOS expression on immune cells

**Figure 3:**
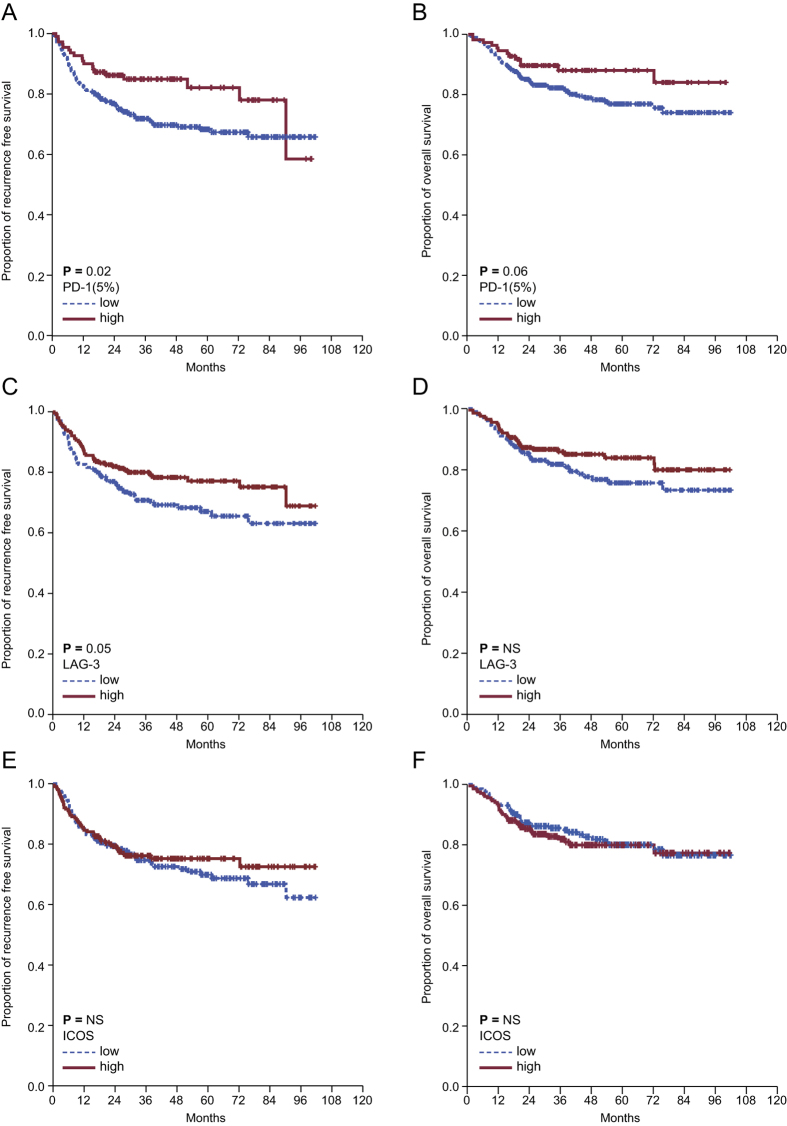
(**A**) The 5-year recurrence-free survival (RFS) rate was 72.4% for PD-L1-positive tumor cells (TC) vs. 65.6% for PD-L1-negative TC; P = 0.206. (**B**) The 5-year overall survival (OS) rate was 80.1% for PD-L1-positive TC vs. 77.7% for PD-L1-negative TC; P = 0.317. (**C**) The 5-year RFS rate was 69.7% for PD-L1-negative immune cells (IC) vs. 80.1% for PD-L1-positive IC; P = 0.005. (**D**) The 5-year OS rate was 75.6% for PD-L1-negative IC vs. 90.6% for PD-L1-positive IC; P = 0.003. (**E**) PD-L1 expression on TC and IC was categorized into four groups according to the percentage of positive cells: TC0 or IC0, 0%; TC1 or IC1, >0% but <5%; TC2 or IC2, ≥5% but <50%; and TC3 or IC3, ≥50%. The counts (frequencies) of TC1/2/3 and IC1/2/3 were 129/402 (32.1%) and 201/402 (50%), respectively, and the overlap between TC1/2/3 and IC1/2/3 was 103/402 (25.6%). The counts (frequencies) of TC2/3 and IC2/3 were 73/402 (18.2%) and 112/402 (27.9%), and the overlap between these two groups was 40/402 (10%). Notably, the counts (frequencies) of TC3 and IC3 were 10/402 (2.5%) and 28/402 (7.0%), respectively, and the overlap between TC3 and IC3 was only 1/402 (0.2%).

We evaluated the expression of immune checkpoint receptors, including PD-1 and LAG-3, and the immune stimulatory receptor ICOS by IC. PD-1, LAG-3, and ICOS expression frequencies on IC were semi-quantitatively scored in each HPF (Fig. 1B(a–f)). PD-1 positivity was defined as PD-1 expression in ≥5% of all IC. Low and high levels of LAG-3 and ICOS in each cell were scored using the median level of tumor infiltration by IC as a cut-off value. The median abundances of LAG-3- and ICOS- expressing IC were 12 and 3 per HPF, respectively. PD-1 positivity appeared to be associated with a lower risk of recurrence and a lower death risk (5-year RFS rate: 82.1% vs. 68.3%, *P* = 0.02; 5-year OS rate: 88.8% vs. 76.9%, *P* = 0.06) (Fig. 3A,B). Multivariate Cox regression analysis demonstrated that patients with more PD-1-expressing IC had superior survival outcomes to those with fewer PD-1-expressing IC (RFS: HR: 0.56, 95% CI: 0.34–0.93, *P* = 0.02; OS: HR: 0.57, 95% CI: 0.31–1.05, *P* = 0.07) (Supplementary Table 1). Patients with a high abundance of LAG-3-expressing IC tended to have prolonged RFS and OS compared to those with a low abundance of LAG-3-expressing IC, although this result did not show statistical significance (Fig. 3C,D). However, ICOS expression did not show a significant prognostic impact on RFS or OS among HNSCC patients (Fig. 3E,F). Of note, PD-1 expression on IC showed a significant positive correlation with LAG-3 and ICOS expression (LAG-3: Pearson correlation coefficient = 0.514, *P* = 0.0001; ICOS: Pearson correlation coefficient = 0.229, *P* = 0.0001).

### PD-L1 expression by immune cells is an independent favorable prognostic factor for RFS and OS among HNSCC patients

As demonstrated in a previous study, PD-L1 is expressed in the cytoplasmic membrane of TC and IC, including T lymphocytes, macrophages, and dendritic cells^2^. As reported in a previous study using the same anti-PD-L1 antibody (clone SP142, Ventana)^2^, we also found that the distribution of PD-L1-positive tumor cells was generally very focal. As tumor-infiltrating macrophages and dendritic cells have a clearly discernible cytoplasm, they displayed a membranous PD-L1staining pattern. As T lymphocytes typically contain scant cytoplasm, PD-L1 staining was detected in a dot-like granular pattern in the perinuclear area. We also noted that PD-L1-positive IC were aggregated in the periphery of tumor cell nests, in stromal bands crossing the tumor cell nests, or as single cells scattered in the stroma or within tumor cell nests, as observed in a previous study^2^ (Supplementary Fig. 3). To clearly distinguish TC from IC in our cases, we performed additional staining with CD3 and cytokeratin in adjacent tumor sections (Supplementary Fig. 4).

**Figure 4:**
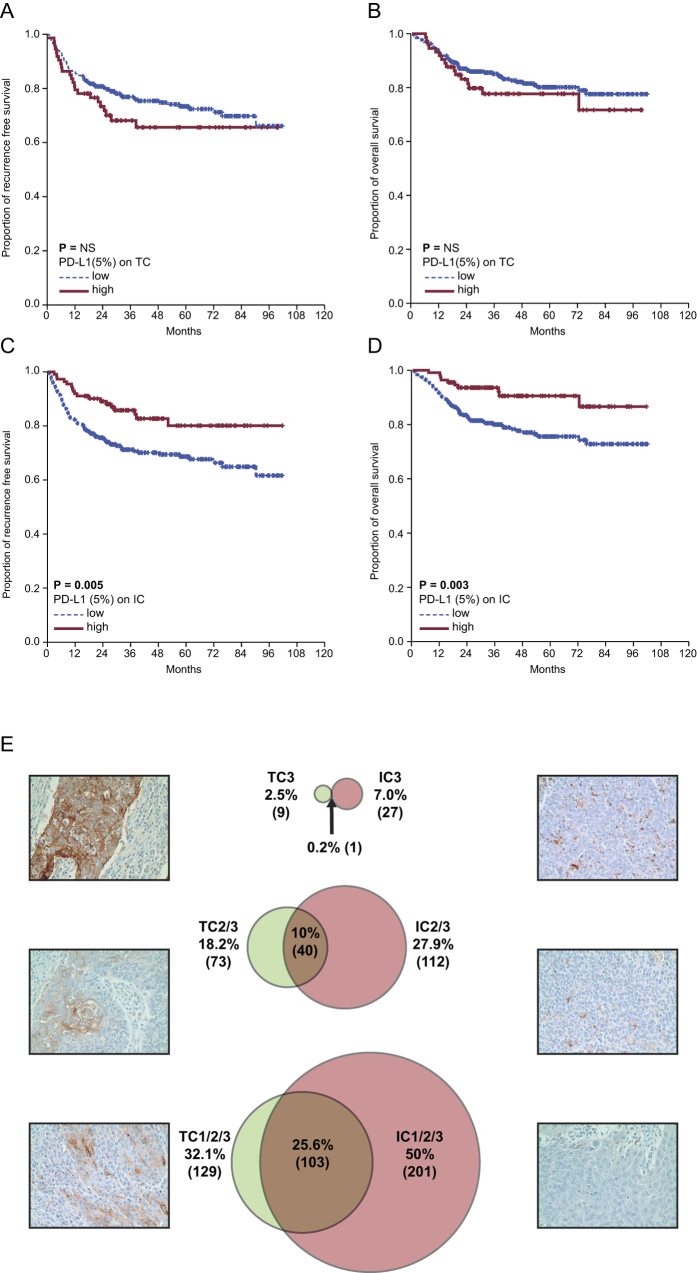
The 402 included patients were assigned to one of two groups based on immune checkpoint expression on T lymphocytes. (**A**) The 5-year RFS rate was 82.1% for PD-1^+^ T cells vs. 68.3% for PD-1^−^ T cells (*P* = 0.02). (**B**) The 5-year OS rate was 88.8% for PD-1^+^ T cells vs. 76.9% for PD-1^−^ T cells (*P* = 0.06). (**C**) The 5-year RFS rate was 77.1% for LAG-3^+^ T cells vs. 67.1% for LAG-3^−^ T cells (*P* = 0.054). (**D**) The 5-year OS rate was 83.9% for LAG-3^+^ T cells vs. 75.7% for LAG-3^−^ T cells (*P* = 0.16). (**E**) The 5-year RFS rate was 75.2% for ICOS^+^ T cells vs. 69.9% for ICOS^−^ T cells (*P* = 0.53). (**F**) The 5-year OS rate was 79.9% for ICOS^+^ T cells vs. 80.0% for ICOS^−^ T cells (*P* = 0.64).

PD-L1 positivity was defined as PD-L1 expression by ≥5% of all TC or IC based on IHC. The frequency of PD-L1 positivity in TC was categorized into four groups, as follows: 273 (67.9%) in 0%, 56 (13.9%) in >0% but <5%, 63 (15.7%) in ≥5% but <50%, and 10 (2.5%) in ≥50% of TC (Fig. 1C(a–d)). The frequency of PD-L1-positive IC was determined using the same criteria: 201 (50%), 89 (22.1%), 84 (20.9%), and 28 (7.0%) in the 0%, >0% but <5%, ≥5% but <50%, and ≥50% groups, respectively (Fig. 1C(e–h)). PD-L1 positivity on TC did not show any significant impact on either RFS or OS compared with PD-L1 negativity on TC (5-year RFS: 72.4% vs. 65.6%, *P* = 0.206; 5-year OS: 80.1% vs. 77.7%, *P* = 0.317) (Fig. 4A,B). In contrast, patients with PD-L1-positive IC had significantly higher 5-year rates of RFS (80.1% *vs*. 69.7%) and OS (90.6% *vs*. 75.6%) (Fig. 4C,D). Multivariate analysis confirmed that patients with PD-L1-positive IC had a significantly lower risk of recurrence and death than those without PD-L1-positive IC after adjusting for sex, smoking, age, pathologic stage, and HPV infection (RFS: AHR 0.49, 95% CI 0.29–0.82, *P* = 0.005; OS: AHR 0.38, 95% CI 0.19–0.73, *P* = 0.04) (Supplemental Table 1). Collectively, these data indicate that high PD-L1 expression on IC is an independent favorable prognostic factor for surgically resected HNSCC. The frequency of infiltration of CD3^+^ and CD8^+^ T lymphocytes was not significantly different between patients with PD-L1-positive and PD-L1 negative IC or TC (CD3^+^ T cells: 85.7 vs. 80.8%, *P* = 0.41; CD8^+^ T cells: 80.4 vs. 78.1%, *P* = 0.71). Therefore, the consideration of PD-L1 expression as a favorable prognostic marker should be restricted to IC, not TC.

When the PD-L1 positivity of IC and TC was analyzed separately, the TC1/2/3 and IC1/2/3 counts (frequencies) were 129 (32.1%) and 201 (50%), respectively, and the overlap between TC1/2/3 and IC1/2/3 was 103 (25.6%). The counts (frequencies) of TC2/3 and IC2/3 were 73 (18.2%) and 112 (27.9%), respectively and the overlap between these two subgroups was 40 (10%). Notably, the TC3 and IC3 counts (frequencies) were 10 (2.5%) and 28 (7.0%), respectively, and only 1 (0.2%) cell showed overlap between TC3 and IC3, reflecting nearly no overlap, as shown in Fig. 4E. This finding suggests that PD-L1 expression might be regulated by distinct mechanisms in TC and IC. There was no significant difference in CD8^+^ or CD3^+^ intensity among TILs between the IC3 and TC3 groups. To analyze the potential differences in PD-L1 expression mechanisms between cell types, transcriptome analysis was performed on tumor samples from the TC3 and IC3 subgroups. Our results suggested that the mechanism underlying the induction of PD-L1 is probably distinct between TC and IC. The results demonstrated that the expression levels of effector T cell markers including IFNγ and GZMB were higher in the IC3 group than in the TC3 group. This result indicated that the expression of PD-L1 on IC3 is regulated by an adaptive IFNγ-mediated mechanism, reflecting pre-existing immunity (Supplementary Fig. 5, Supplementary Table 2).

### HPV

We divided the 402 HNSCC patients into two groups according to the presence or absence of oncogenic HPV infection (as demonstrated by p16 expression) in their tumors. The frequencies of HPV positivity were 41.8% (168/402) among all HNSCC patients and 80.3% (98/122) among oropharyngeal cancer (OPC) patients. Based on Kaplan-Meier estimates, the survival outcomes of patients with HPV-positive cancer were significantly better than those of patients with HPV-negative cancer (5-year RFS: 82.4% vs. 64.3%, *P* < 0.001; 5-year OS: 85.6% vs. 75.3%, *P* = 0.006). Patients with HPV-positive OPC also had significantly better RFS and OS than those with HPV-negative OPC. HPV-positive OPC samples were more heavily infiltrated by CD3^+^ cells (*P* < 0.001), CD8^+^ cells (*P* = 0.001), PD-1^+^ TILs (*P* = 0.01), and LAG-3^+^ TILs (*P* = 0.001) than HPV-negative OPC samples. The trend of increased infiltration in HPV-positive OPC samples was also observed in the form of high levels of Foxp3^+^ cell infiltration. PD-L1-expressing IC were more frequently observed in HPV-positive OPC than in HPV-negative OPC (Table 2). HPV-positive OPC samples were infiltrated by many PD-L1^+^ IC, PD-1^+^ TIL, CD3^+^ cells, and the presence of CD8^+^ cells correlated with better OS. Indeed, patients with PD-L1-positive IC had an overall 5-year OS rate of 93.8%, whereas patients with PD-L1-negative IC had an overall 5-year OS rate of 81.4% (*P* = 0.04). HPV-positive OPC patients displaying high CD8^+^ cell infiltration exhibited superior RFS and OS to those displaying low CD8^+^ cell infiltration (5-year RFS rate: 91.0% vs. 69.0%, *P* < 0.001; 5-year OS rate: 94.2% vs. 72.1%, *P* < 0.001) In contrast, the levels of infiltration by Foxp3^+^ T cells, PD-1^+^ TILs, and LAG-3^+^ TILs did not correlate with the OS of HPV-positive OPC patients.

## Discussion

As immunotherapy is highlighted as an effective treatment strategy for HNSCC patients, interest in immune-related biomarkers has greatly increased. Although CD3^+^ or CD8^+^ TIL infiltration is known to correlate with favorable prognosis among HNSCC patients, much remains unknown regarding immune-related biomarkers of HNSCC. In the present study, we comprehensively evaluated the frequency of PD-L1 expression in TC and IC. Additionally, the expression of other immune regulatory receptors (i.e., PD-1, ICOS, and LAG-3), the abundance of several types of T cells (i.e., CD3^+^, CD8^+^, and T_reg_) and their prognostic impacts on resected HNSCC outcomes were evaluated. Patients with PD-L1 expression on IC had favorable survival outcomes, whereas PD-L1 expression on TC or in the whole tissue section, including both TC and IC, did not show any prognostic impact on survival outcome among HNSCC patients. In our study, high abundances of CD3^+^ or CD8^+^ TILs were independently associated with prolonged survival outcomes among HNSCC patients, and our findings are consistent with those of previous reports^9^. CD8^+^ T cells are predominantly cytotoxic T cells, a crucial component of the cellular immune system, and are pivotal for cell-mediated anti-tumor immune responses^20^,^29^,^30^.

Most importantly, we comprehensively evaluated PD-L1 expression on IC, TC, and whole tissue sections. In whole tissue sections, regardless of TC or IC, PD-L1 expression did not demonstrate any prognostic impact for the included patients (data not shown). Surprisingly, only PD-L1 expression on IC showed a favorable prognostic impact among these HNSCC patients. The prognostic value of PD-L1 remains controversial in HNSCC patients. Several reports have shown that PD-L1 expression is a negative prognostic factor in several cancer types, including renal, colorectal, and lung cancers^16^,^17^,^18^, but others have reported that PD-L1 is a favorable prognostic factor in metastatic melanomas, NSCLC, and Merkel cell carcinomas^21^,^22^,^31^. Recently, it has been reported that PD-L1 expression and TIL abundance were independent favorable prognostic factors for OS and RFS in laryngeal squamous cell carcinoma patients^20^. To date, no comprehensive studies of IC and TC have focused on HNSCC. In the majority of related studies, the expression of PD-L1 in tumor sections has been evaluated without fine discrimination between IC and TC. Based on our results, PD-L1 expression on TC and on IC might have different prognostic values and might be regulated by distinct mechanisms. When we separately analyzed PD-L1 positivity on TC and IC, which was categorized into four groups, PD-L1 was exclusively expressed on either TC or IC. Of note, TC3 and IC3 represent distinct populations with <1% overlap, suggesting that PD-L1 might be regulated by distinct mechanisms between the subsets of TC and IC. Based on our transcriptome analysis results, PD-L1 expression on IC is regulated via adaptive mechanisms and reflects pre-existing immunity. However, PD-L1 expression on TC could be regulated by tumor-intrinsic mechanisms including HIF1-α induction by hypoxia, oncogenic signaling pathway activation, or epithelial-mesenchymal transition, although we could not identify the distinct gene signature in TC3^32^,^33^,^34^,^35^. Collectively, our results highlighted the importance of comprehensive assessment of PD-L1 expression in both TC and IC. Notably, there was no difference in lymphocyte infiltration between the PD-L1 positive TC and IC populations. After compensation for T cell infiltration, PD-L1 positivity of IC remained a favorable prognostic factor in this HNSCC patient cohort, and this result suggests its independent favorable prognostic value.

Notably, high PD-1 expression was associated with prolonged survival outcomes among resected HNSCC patients. This result is consistent with recently reported data in HPV-positive HNSCC patients^36^, in which those with a high level PD-1-expressing T cells correlated with better survival outcome than those with a low level of these cells^36^. Additionally, in follicular lymphoma, a high degree of infiltration by PD-1-positive T cells might be related to a favorable outcome and a reduced risk of transformation. In contrast, several other studies have found that PD-1 expression on IC correlated with poor prognosis and was associated with shorter survival among renal cell cancer, nasopharyngeal cancer, and Hodgkin lymphoma patients^37^,^38^,^39^. PD-1 on T cells and IC is known to perform an inhibitory function against effector T cells, and PD-1 is highly upregulated in exhausted T cells. Several hypotheses could explain this paradoxical favorable prognosis of PD-1 expression on IC. Given that the increase in PD-1 expression could be the result of T cell receptor activation, PD-1 might remain upregulated in the context of persistent antigen-specific immune stimulation. Moreover, PD-1-expressing T cells might include tumor antigen-specific T cells that exert anti-tumor effects in the tumor microenvironment. High expression of PD-1 more closely reflects an “exhausted status” than intermediate or low PD-1 expression. However, we could not analyze the levels of PD-1 expression in detail due to the limitations of IHC and consequent difficulties in determining the baseline PD-1 staining intensity. Thus, we could not determine the precise activating status of PD-1 expression on T cells. We can provisionally conclude that HNSCC patients with PD-1 positive T cells had favorable survival outcomes because tumor antigen-specific T cells are present among PD-1 positive T cells. In a pooled analysis including advanced NSCLC patients who were pre-screened and/or enrolled in three trials evaluating the anti-PD-L1 antibody atezolizumab (PCD4989g Phase Ia; FIR Phase II; POPLAR Phase II; 1,273 patients), high expression of the receptors PD-1 and B7.1 was associated with improvement in OS following treatment with atezolizumab. Taken together, this evidence suggests that patients with high expression of PD-1 on TILs could be potential candidates for anti-PD-1/PD-L1 blockade.

High expression of LAG-3 on IC tended to prolong survival in our study, although this result lacked statistical significance. This observation suggests that LAG-3-expressing IC might include tumor antigen-specific T cells. ICOS did not show any prognostic impact in our cohort. However, because of the low frequencies of LAG-3- and ICOS-expressing TILs, we cannot draw a conclusion regarding their prognostic impact. As expected, LAG-3 and ICOS expression appears to be strongly related to PD-1 expression on TIL. Our study demonstrated that high levels of Foxp3^+^ T_reg_s in HNSCC patients positively correlated with better prognosis^40^. These results concerning the favorable prognostic value of intra-tumoral T_reg_s in HNSCC have subsequently been reproduced by various groups^36^. Based on correlation analysis between Foxp3^+^ TILs and PD-L1 expression on either IC or TC, PD-L1 expression on IC and TC was significantly associated with an increased frequency of Foxp3^+^ TIL in our cohort. This phenomenon might be because PD-L1 and T_reg_ cells were also recruited under immuno suppressive conditions such as the tumor microenvironment. Thus, there could be positive correlations between Foxp3^+^ TILs and PD-L1 expression on either IC or TC. A recent study reported that Foxp3^+^ T_reg_ cells are divided into specific subsets including the highly differentiated and most suppressive Foxp3^+^ high T_reg_ cells^41^. We do not know the exact status of T_reg_ cells in previous reports including ours, because previous studies typically used Foxp3 as a representative marker of T_reg_ cells. HPV-positive OPC was more heavily infiltrated by CD3^+^ and CD8^+^ T cells and by PD-1^+^ and LAG-3^+^ IC than HPV-negative OPC. Moreover, PD-L1 expression on IC was more frequently observed in HPV-positive OPC than in HPV-negative OPC and showed independent, favorable prognostic impact on survival outcomes in HPV-positive OPC patients. Unfortunately, we could not identify whether various immune markers can predict the response to immune checkpoint blockade because our cohort did not include patients treated with this therapy. In the future, we must evaluate the value of predictive immune markers in HNSCC patients treated with immunotherapy.

In summary, our study demonstrated that PD-L1 expression on IC, not on TC, the abundance of PD-1 expressing IC, and the abundance of Foxp3^+^ T_reg_ cells are independent predictors of favorable OS in resected HNSCC patients. Assessment of the expression of immune-related molecules in HNSCC could produce evidence relevant to the appropriateness of treatment via immune checkpoint blockade. Moreover, our findings highlight the importance of comprehensive assessment of both TC and IC in HNSCC.

## Additional Information

**How to cite this article**: Kim, H. R. *et al*. PD-L1 expression on immune cells, but not on tumor cells, is a favorable prognostic factor for head and neck cancer patients. *Sci. Rep.* **6**, 36956; doi: 10.1038/srep36956 (2016).

**Publisher’s note**: Springer Nature remains neutral with regard to jurisdictional claims in published maps and institutional affiliations.

## Supplementary Material

Supplementary Information

## Figures and Tables

**Table 1:** Baseline characteristics.

**Characteristics**	**Total (n = 402) n (%)**
*Age, years*	
Median (range)	58 (22–88)
*Sex*	
Male	302 (75.1)
Female	100 (24.9)
*Primary sites*^§^	
Oral cavity	204 (50.7)
Oropharynx	122 (30.3)
Larynx	44 (10.9)
Hypopharynx	28 (7)
Nasal cavity	4 (1.0)
*Differentiation*	
Well differentiated	149 (37.1)
Moderately differentiated	200 (49.8)
Poorly differentiated	53 (13.2)
*pT stage*	
T1	183 (45.5)
T2	148 (36.8)
T3	28 (7.0)
T4	43 (10.7)
*pN stage*	
N0	180 (44.8)
N1	76 (18.9)
N2	146 (36.1)
N3	3 (0.7)
*AJCC7 stage*	
Stage I	116 (28.9)
Stage II	43 (10.7)
Stage III	74 (18.4)
Stage IVA/B	169 (42.0)
*Smoking*	
Never smoker	156 (38.9)
Former smoker*	81 (20.1)
Current smoker	165 (41.0)
Smoking history (pack*years)	
Median (range)	18 (0–100)
*Resection margin*	
Positive	94 (23.4)
Negative	308 (76.6)
*Lymphovascular invasion*	
Yes	76 (18.9)
No	326 (81.1)
*Perineural invasion*	
Yes	54 (13.4)
No	348 (86.6)
*p16 expression status*	
Positive	163 (40.5)
Negative	239 (59.5)
*Adjuvant treatment*	
CCRT	101 (25.3)
Radiotherapy	151 (37.8)
Chemotherapy	2 (0.5)
No	146 (36.5)

**Table 2:** Correlations of HPV infection status with T-cell infiltration and PDL-1 expression.

		**HPV-positive oropharyngeal cancer (n = 98)**	**HPV-negative oropharyngeal cancer (n = 24)**	**P-value**
CD3 + TIL	High	73 (59.8%)	5 (4.1%)	<0.001
	Low	25 (20.5%)	19 (15.6%)	
CD8 + TIL	High	73 (59.8%)	9 (7.4%)	0.001
	Low	25 (20.5%)	15 (12.3%)	
Foxp3 + T cell	High	53 (43.4%)	8 (6.6%)	0.055
	Low	45 (36.9%)	16 (13.1%)	
PD-L1 + tumor cell	High	19 (15.6%)	3 (2.5%)	0.324
	Low	79 (64.8%)	21 (17.2%)	
PD-L1 + immune cell	High	45 (36.9%)	4 (3.3%)	0.01
	Low	53 (43.4%)	20 (16.4%)	
PD-1 TIL	High	48 (39.3%)	3 (2.5%)	0.01
	Low	50 (41.0%)	21 (17.2%)	
LAG-3 TIL	High	73 (59.8%)	6 (4.9%)	0.001
	Low	25 (20.5%)	18 (14.8%)	
ICOS TIL	High	39 (32.0%)	12 (9.8%)	0.248
	Low	59 (48.4%)	12 (9.8%)	

Abbreviations: HPV, human papilloma virus; TIL, tumor infiltrating lymphocyte.
