# Supplementary material for: PD-L1 expression on immune cells, but not on tumor cells, is a favorable prognostic factor for head and neck cancer patients
Source: Sci Rep. 2016 Nov 14;6:36956. doi: 10.1038/srep36956 (PMC5107906; doi:10.1038/srep36956)

Supplementary Files

PD-L1 expression on immune cells, but not on tumor cells, is a favorable prognostic factor for head and neck cancer patients

Hye Ryun Kim[1], Sang-Jun Ha[2], Min Hee Hong[1], Su Jin Heo[1], Yoon Woo Koh[3], Eun Chang Choi[3], Eun Kyung Kim[4], Kyoung Ho Pyo[5], Inkyung Jung[6], Daekwan Seo[7], Jaewoo Choi[7], Byoung Chul Cho[1,5]* and Sun Och Yoon[4]*

[1]Yonsei Cancer Center, Division of Medical Oncology, Yonsei University College of Medicine, Seoul, Korea; [2]Department of Biochemistry, College of Life Science & Biotechnology, Yonsei University, Seoul, Korea; [3]Department of Otorhinolaryngology, Yonsei University College of Medicine, Seoul, South Korea; [4]Department of Pathology, Yonsei University College of Medicine, Seoul, Korea; [5]JE-UK Institute for Cancer Research, JEUK Co., Ltd., Gumi-City, Kyungbuk, Korea; [6]Department of Biostatistics and Medical Informatics, Yonsei University College of Medicine, Seoul, Korea; [7]Severance Biomedical Science Institute, Yonsei University of College of Medicine, Seoul, Korea

Supplementary Files

Supplementary Table (number 2)

Supplementary Figure (number 5)

Supplementary Table 1 Multivariate Cox regression analysis for the prediction of RFS and OS

	RFS			OS		
	***P*-value**	**HR**	**95% CI**	***P*-value**	**HR**	**95% CI**
Age (<58 vs. ≥58 years)	0.017	1.61	1.088 - 2.383	0.017	1.782	1.109 - 2.862
Tumor stage	<0.001	1.38	1.166 - 1.635	<0.001	1.716	1.361 - 2.165
Smoking	0.034	1.265	1.018 - 1.572	0.264	1.157	0.896 - 1.495
p16 positivity	<0.001	0.416	0.269 - 0.644	0.007	0.497	0.3 - 0.824
PD-L1 on tumor cells	0.208	1.346	0.848 - 2.137	0.319	1.325	0.762 - 2.306
PD-L1 on immune cells	0.005	0.487	0.293 - 0.809	0.04	0.383	0.197 - 0.746
PD-1 on TILs	0.024	0.564	0.343 - 0.926	0.07	0.574	0.316 - 1.045
$CD3^+$ T cells	0.005	0.573	0.387 - 0.849	0.017	0.561	0.35 - 0.901
$CD8^+$ T cells	<0.001	0.469	0.314 - 0.701	0.003	0.484	0.299 - 0.784
$Foxp3^+$ T cells	0.006	0.577	0.389 - 0.854	0.096	0.674	0.424 - 1.072
$LAG3^+$ T cells	0.055	0.685	0.466 - 1.008	0.163	0.72	0.454 - 1.142
$ICOS^+$ T cells	0.531	0.885	0.603 - 1.298	0.646	1.113	0.705 - 1.759

Abbreviation: RFS, recurrence-free survival; OS, overall survival; HR, hazard ratio; CI, confidence interval

Supplementary Table 2. List of genes highly expressed in the IC3 group compared to the TC3 group

**Gene symbol**	**P-value**	**Fold change in expression**	**Description**
CD8A	0.018	2.28	T_{eff} signature genes
GZMA	0.059	1.90	
GZMB	0.152	1.21	
IFNγ	0.233	1.31	
EOMES	0.196	1.31	
PRF1	0.360	0.72	
CXCL9	0.323	1.10	
TBX21	0.115	1.40	
CD3E	0.007	3.28	Positive regulation of lymphocyte proliferation
CD74	0.0005	2.40	Antigen processing and presentation of peptide antigens via MHC class II molecules
CTSS	0.001	3.16	
FCER1G	0.001	2.49	
HLA-DOA	0.001	2.55	
HLA-DQA1	0.009	4.49	
JAK3	0.011	3.11	B cell activation
BANK1	0.009	3.45	
IGLL5	0.012	3.21	
PTPRC	0.012	3.13	
TNFRSF13C	0.016	3.62	
BCL2	0.119	3.42	

Supplementary Figure 1.
Survival outcomes at each disease stage among the 402 head and neck squamous cell cancer patients.
(A) Recurrence-free survival of the patients according to disease stage. (B) Overall survival of the patients according to disease stage.

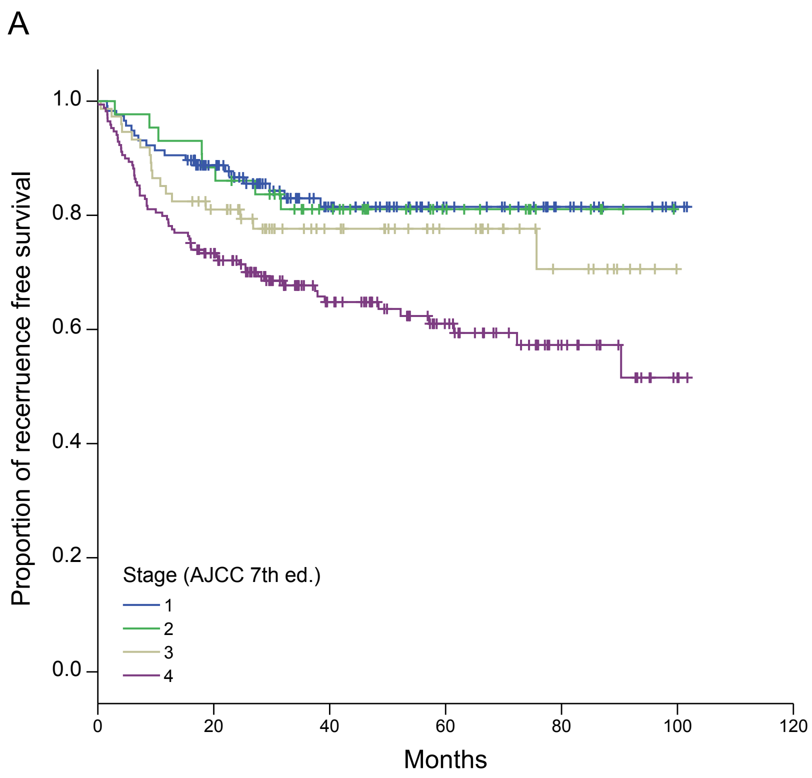

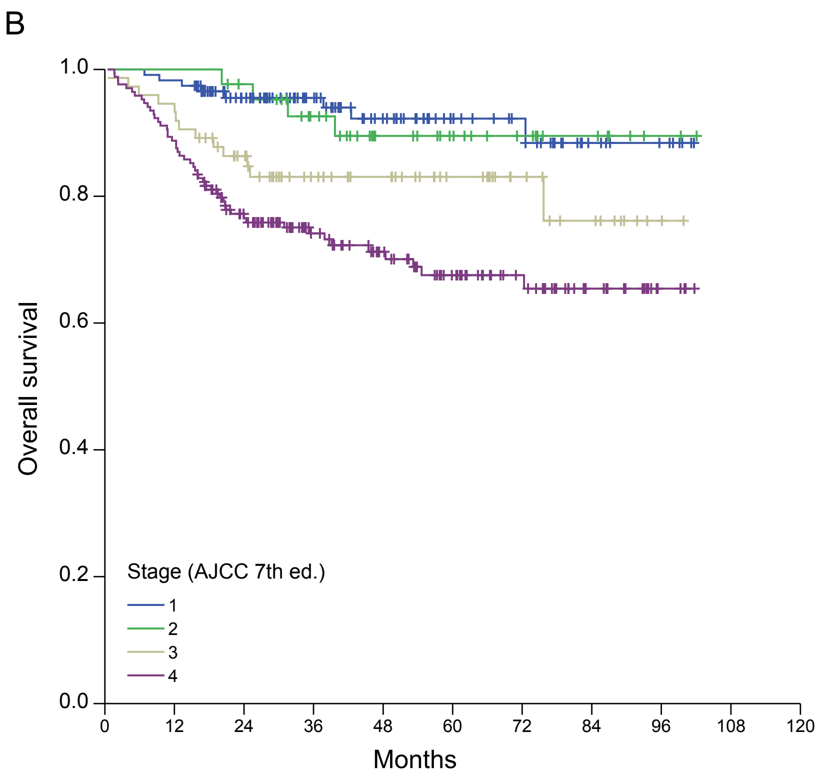

Supplementary Figure 2.
The frequency of (A) CD3+ tumor−infiltrating lymphocytes (TILs), (B) CD8+ TILs, (C) Foxp3+ TILs, (D) LAG3+ TILs, and (E) ICOS+ TILs. The X−axis represents the average number of cells in five representative high−power fields under 400 x magnifications, and the y−axis represents the frequency of the corresponding cell type.

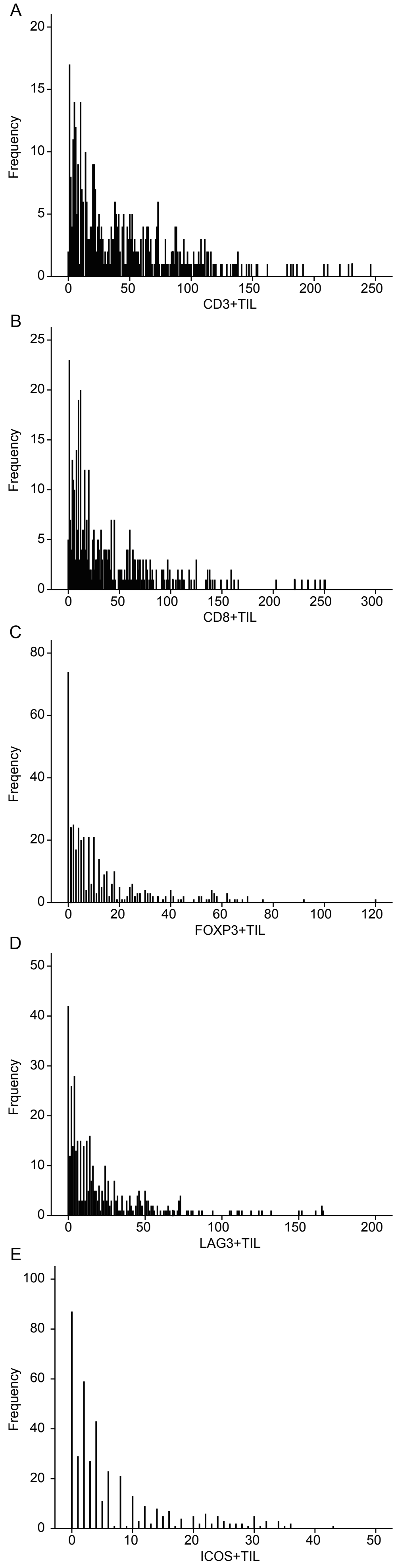

Supplementary Figure 3.
The distribution of PD-L1+ tumor cells (TC) was generally very focal (black arrow; A). Tumor-infiltrating T lymphocytes variably expressed PD-L1, and PD-L1-positive T cells were scattered within tumor cell nests (dotted arrow; A) or were aggregated along the periphery of tumor cell nests (dotted arrow; B). At high magnification, PD-L1 was found to be expressed by TC in intracytoplasmic and membranous patterns (C; black arrow) and by T cells in a perinuclear, dot-like, granular pattern (D; dotted arrow). A portion of tumor-infiltrating macrophages also variably expressed PD-L1 in intracytoplasmic and membranous patterns (E; empty arrow). The outlines of tumor nests are highlighted by dotted lines. Figures were captured at 200X magnification (A-B) or 400X magnification (C-E)

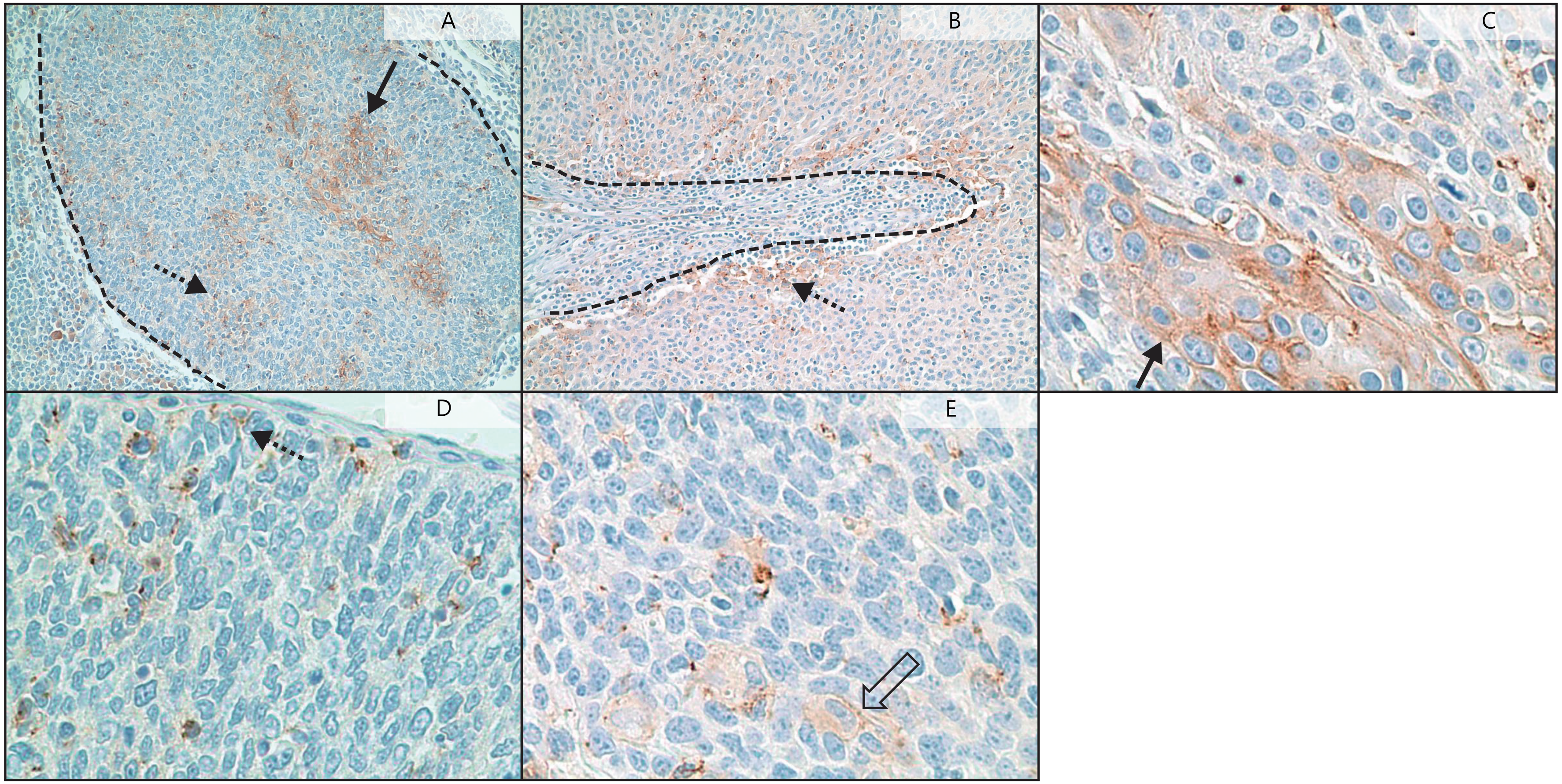

Supplementary Figure 4.
Tumor infiltrating T cells (A, B, and C: empty arrow) are noted as non-cohesive scattered small cells having round dense nuclei and clear cytoplasm, and these histologic features of T cells are discernable from the cancer cells which show larger coarse nuclei, granular cytoplasm, and cohesive growth (B and C). When the cytoplasm of tumor infiltrating CD3+ T cells were negative for PD-L1 in within the tumor cells showing diffuse strong PD-L1 expression (B), these contrasted expression pattern of PD-L1 was easily discernible because of the histologic characteristics of each cell type.

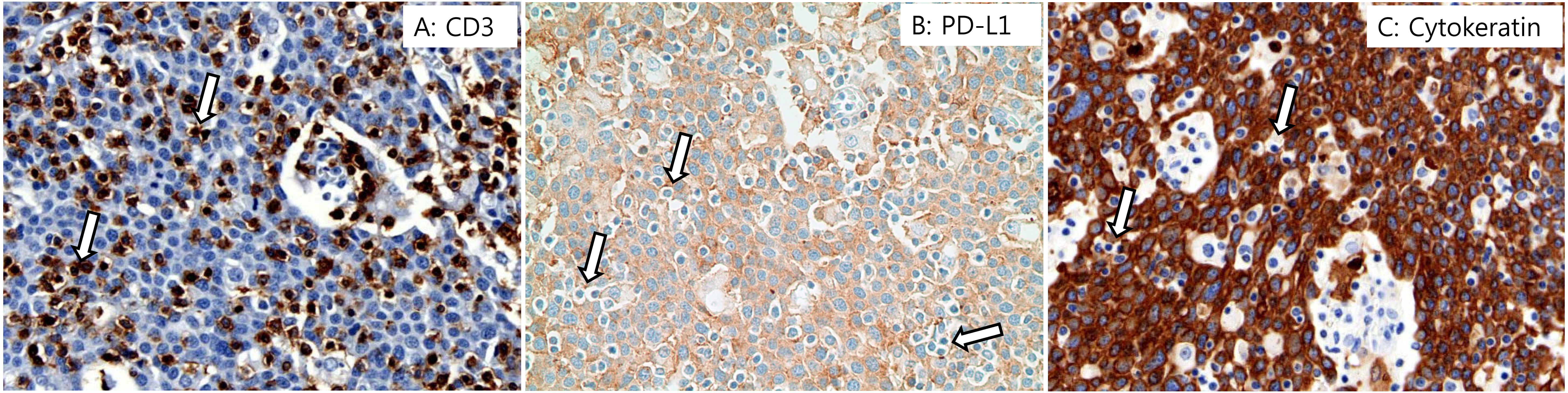

Supplementary Figure 5.

IC3 tumors are characterized by high expression of the Teff signature, which was defined by the expression of CD8A, GZMA, GZMB, IFN γ, EOMES, PRF1, CXCL9, and TBX21. P values are presented for TC3 tumors vs. IC3 tumors. STD, standardized; Teff, effector T cell; FC, fold change.

T_{eff} signature

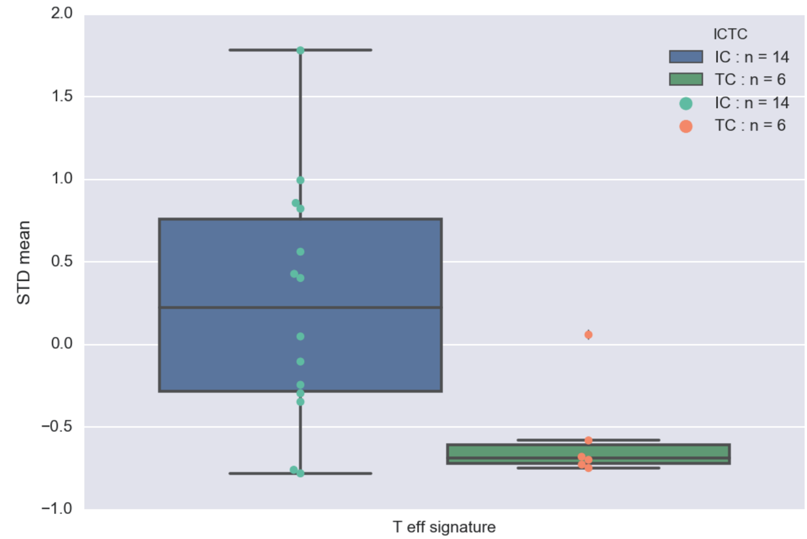

CD8A
P= 0.01, FC= 2.28

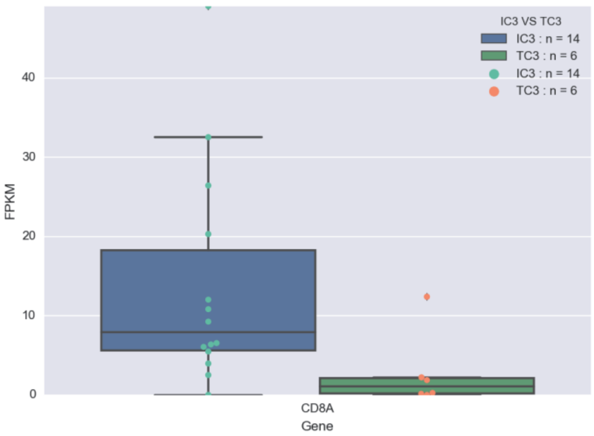

GZMB
P= 0.15, FC=1.21

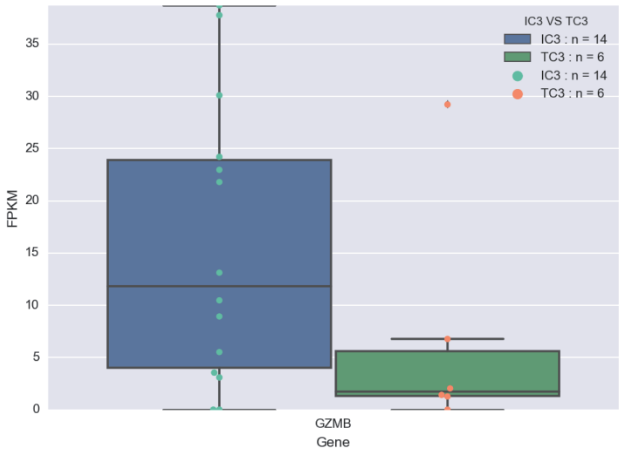

IFNγ
P=0.23, FC=1.31

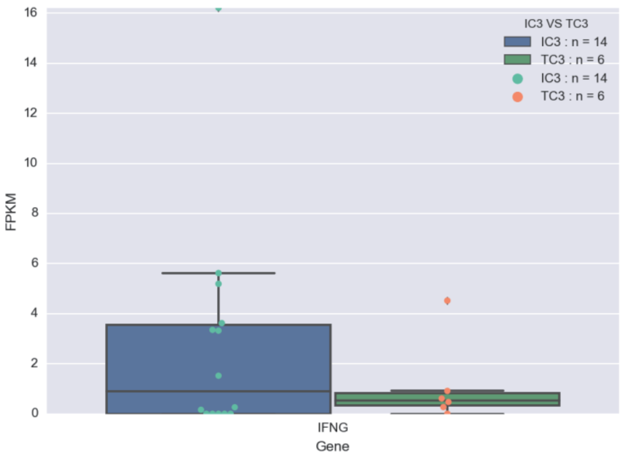

Supplement: Supplementary Information [file srep36956-s1.pdf]
